# High Expression of Glycolytic Genes in Cirrhosis Correlates With the Risk of Developing Liver Cancer

**DOI:** 10.3389/fcell.2018.00138

**Published:** 2018-10-31

**Authors:** Nathan C. W. Lee, Maria Annunziata Carella, Salvatore Papa, Concetta Bubici

**Affiliations:** ^1^Cell Signaling and Cancer Laboratory, Leeds Institute of Cancer and Pathology, Faculty of Medicine and Health, University of Leeds, St James’s University Hospital, Leeds, United Kingdom; ^2^Division of Biosciences, Department of Life Sciences, College of Health and Life Sciences, Institute of Environment, Health and Societies, Brunel University London, Uxbridge, United Kingdom; ^3^Faculty of Medicine, Imperial College London, London, United Kingdom

**Keywords:** glucose metabolism, aerobic glycolysis, the Warburg effect, liver, liver cancer

## Abstract

A marked increase in the rate of glycolysis is a key event in the pathogenesis of hepatocellular carcinoma (HCC), the main type of primary liver cancer. Liver cirrhosis is considered to be a key player in HCC pathogenesis as it precedes HCC in up to 90% of patients. Intriguingly, the biochemical events that underlie the progression of cirrhosis to HCC are not well understood. In this study, we examined the expression profile of metabolic gene transcripts in liver samples from patients with HCC and patients with cirrhosis. We found that gene expression of glycolytic enzymes is up-regulated in precancerous cirrhotic livers and significantly associated with an elevated risk for developing HCC. Surprisingly, expression levels of genes involved in mitochondrial oxidative metabolism are markedly increased in HCC compared to normal livers but remain unchanged in cirrhosis. Our findings suggest that key glycolytic enzymes such as hexokinase 2 (HK2), aldolase A (ALDOA), and pyruvate kinase M2 (PKM2) may represent potential markers and molecular targets for early detection and chemoprevention of HCC.

## Introduction

Hepatocellular carcinoma (HCC) is the main type of primary liver cancer and the second leading cause of cancer-related mortality worldwide, with more than 700,000 deaths every year (El-Serag, 2011; Jemal et al., 2011; Forner et al., 2018). The incidence of HCC has risen considerably over the last two decades, especially in United States and Europe (El-Serag, 2011; Jemal et al., 2011). At present, treatment options mainly consist of tumor resection, liver transplant, chemotherapies and radiologic intervention, all of which are limited to patients with early-stage disease. However, the large majority of HCC patients are usually diagnosed at advanced stages, which lack of curative therapies (Llovet et al., 2008; Ulahannan et al., 2014; Forner et al., 2018). Therefore, early HCC detection and prevention are required for reducing the high mortality rate. A better understanding of the molecular basis of HCC formation and the identification of markers are essential for the development of preventive therapies targeting the specific HCC-promoting factors and thereby improvement prognosis.

The development of HCC has been closely linked to cirrhosis, an inflammatory liver condition in which the normal liver tissue is replaced by scar tissue and regenerative nodules after long-term damage induced by various etiologies including hepatitis B (HBV) or C (HCV) viral infection, chronic alcohol consumption or nonalcoholic fatty liver disease (Wright, 1991; Borzio et al., 1995; Donato et al., 2001). In fact, up to 90% of all cases of HCC develop in patients with cirrhosis, suggesting a role for this liver condition in the process of hepatocarcinogenesis (El-Serag, 2011; Jemal et al., 2011; Forner et al., 2018). At the cellular level, the occurrence of cirrhosis is tightly coupled with multiple rounds of hepatocyte death, inflammatory responses and compensatory hepatocyte proliferation, resulting in the formation of the regenerative nodules surrounded by fibrous bands characteristic of cirrhotic livers. It is now clear that the regenerative nodules consist of a mixed population of proliferative progenitor cells, newly generated hepatocytes and apoptosis-resistant (damaged) hepatocytes that, overtime, are likely to develop into dysplastic nodules, leading to the development of HCC (Borzio et al., 1995; Schuppan and Afdhal, 2008). Therefore, cirrhosis is recognized as a precancerous state and is important for the investigations of molecular markers of HCC development and preventive strategies. Although the etiology and pathological characteristics of liver cirrhosis have been reported in fully, the underlying molecular mechanisms of its progression to HCC are far less unknown.

Like many other cancers, HCC develops slowly after progressive accumulation of genetic and epigenetic alterations in liver cells accompanied by substantial changes in energy metabolism leading to unrestricted proliferation of mature hepatocytes. One of the most common metabolic changes observed in HCC cells is an increase in the rate of glycolysis with consequent lactate production (Kitamura et al., 2011; Huang et al., 2013; Iansante et al., 2015; reviewed in Hay, 2016). This metabolic phenomenon, known as the Warburg effect or aerobic glycolysis, occurs even in the presence of copious levels of oxygen and functional mitochondria (Gatenby and Gillies, 2004; Lunt and Vander Heiden, 2011; DeBerardinis and Chandel, 2016; Liberti and Locasale, 2016). Indeed, under aerobic conditions, normal differentiated hepatocytes typically metabolize glucose into pyruvate, which is further metabolized to carbon dioxide in the mitochondria through the tricarboxylic acid (TCA) cycle and oxidative phosphorylation (OXPHOS) for ATP production, whereas under low oxygen conditions, the hepatocytes convert the pyruvate derived from glycolysis into lactate. Thus, HCC cells seem to re-adjust their energy metabolism by shifting toward glycolysis irrespective of oxygen availability in a manner similar to virtually all cancer cells (Iansante et al., 2015; Hay, 2016). This metabolic shift allows the highly proliferating HCC cells to accumulate intermediary glucose metabolites that can be channeled into biosynthetic pathways such as the pentose phosphate pathway (PPP), which generates the cellular reductant NADPH and macromolecules (nucleotides, amino acids, and fatty acids) required for the doubling of biomass, and to suppress apoptosis (Jones and Thompson, 2009; Hay, 2016; Kowalik et al., 2017). There is ample evidence that the readjustment of cell metabolism occurs as a consequence of activation of oncogenes or loss of tumor suppressors that influences the expression and activities of metabolic enzymes to stimulate glucose consumption (Hay, 2016). Moreover, an increased rate of glucose uptake, which is due to an up-regulation of the main glucose transport GLUT1, is also a remarkable feature observed in HCC cells (Hay, 2016). Nevertheless, limited data are available on altered gene expression of the enzymes involved in glycolysis and oxidative mitochondrial metabolism *in vivo*. In addition, whether the aerobic glycolytic metabolism is operative in the early stage of the hepatic carcinogenesis particularly in cirrhosis and correlates with poor patient prognosis have not been completely elucidated. Such a background prompted us to investigate the expression level of enzymes required for the glycolytic and mitochondrial metabolism in liver samples from patients with cirrhosis and patients with HCC available from six open source data sets.

## Materials and Methods

### Data Sets Review

Differential gene expression analyses involving multiple clinical samples were performed using different data sets available through Gene Expression Omnibus (GEO) database (Barrett, 2013). Independent of age, gender, race, and region, we selected one HCC data set (GSE36376) and three cirrhotic data sets (GSE25097, GSE6764, and GSE14323) (accessed on March–May 2018) (Wurmbach et al., 2007; Sung et al., 2012; Lim et al., 2013; Levy et al., 2016) as they contained the largest cohort of HCC livers compared to adjacent non-tumor livers and cirrhotic livers compared to normal healthy livers, respectively. The GSE36376 data set consists of tumor and adjacent non-tumor liver tissues containing no necrosis or hemorrhage from 240 primary HCC patients who were treated with surgical resection or liver transplantation. None of the patients received preoperative chemotherapy (Lim et al., 2013). The GSE25097 data set consists of six liver specimens from healthy donors and 40 cirrhotic livers (Sung et al., 2012). The GSE6764 data set consists of 13 HCV-associated cirrhotic liver tissues compared with healthy livers of 10 patients undergoing resection: one patient for hepatic haemangioma, three for focal nodular hyperplasia, two for adenoma/cystadenoma, one for neuroendocrine tumor, and one living donor liver transplantation (Wurmbach et al., 2007). The GSE14323 data set consists of 41 HCV-associated cirrhotic liver tissues compared with healthy livers of 18 patients (Levy et al., 2016). For HCC, we also used a second independent data set from The Cancer Genome Atlas-Liver Hepatocellular Carcinoma (TCGA-LIHC) database, which contained a cohort of more than 350 HCC samples compared to 50 normal healthy livers (Ally et al., 2017). The TCGA-LIHC data set consists of surgical resection of biopsy biospecimens collected from patients diagnosed with HCC, and had not received prior treatment for their disease (ablation, chemotherapy, or radiotherapy). Each frozen primary tumor specimen had a companion normal tissue specimen (blood or blood components, including DNA extracted at the tissue source site). Pathology quality control was performed on each tumor and normal tissue specimen. Haematoxylin and eosin (H&E) stained sections from each sample were subjected to independent pathology review to confirm that the tumor specimen was histologically consistent with the allowable HCCs and the adjacent tissue specimen contained no tumor cells. Adjacent tissue with cirrhotic changes was not acceptable as a germline control (Ally et al., 2017). The data set GSE15654 was used for the HCC risk-association of cirrhotic patients. The data set consists of 216 livers from patients with hepatitis C-related early-stage (Child-Pugh class A) cirrhosis who were prospectively followed up for a median of 10 years (Hoshida et al., 2013). Clinicopathological features of each cohort analyzed in this study are shown in the relevant cited studies.

### Data Sets Analyses

For the analyses of GEO data sets, the raw data of each data set was downloaded from GEO accession links. Samples of each data set were categorized into two groups: diseased patients versus control tissue. The differential expression of genes between the two groups was calculated using a non-parametric Mann–Whitney *U* test, assigning a specific threshold (*P*-value <0.05). The analyses were performed in Prism GraphPad packages, as previously reported (Barbarulo et al., 2013).

For the differential gene expression analyses of TGCA-LIHC we used a user-friendly, interactive web resource for analyzing cancer transcriptome data accessible at http://ualcan.path.uab.edu/index.html (accessed on April/May 2018) (Chandrashekar et al., 2017). For the overall survival plots of the TGCA-LIHC data set we used GEPIA, a newly developed interactive web server available at http://gepia.cancer-pku.cn/index.html (accessed on April/May 2018) (Tang et al., 2017). KM plotter (available at http://kmplot.com/analysis/index.php?p=service&cancer=liver_rnaseq) (accessed on August 2018) was used to generate Kaplan-Meyer plots of an additional liver HCC data set (Szász et al., 2016). In both cases, the median value of gene expression was used to arrange the low and high expression groups. The overall survival significance between the two groups was calculated using Log-rank test, assigning a specific threshold (*P*-value <0.05).

## Results

A salient feature of HCC cells is that they adjust their metabolic profile to fulfill the bioenergetics and anabolic demands of the high rates of proliferation (Kitamura et al., 2011; Huang et al., 2013; Iansante et al., 2015; Hay, 2016). Yet, little is known about the metabolic changes at premalignant stages of disease. To have a complete overview of the metabolic genes expressed in HCC and premalignant stages of disease, we analyzed the transcription profiling of enzymes involved in glycolysis, PPP, TCA, and OXPHOS in liver samples from patients with HCC and patients with cirrhosis.

### Gene Expression of Glycolysis and PPP Enzymes in HCC

**FIGURE 1:**
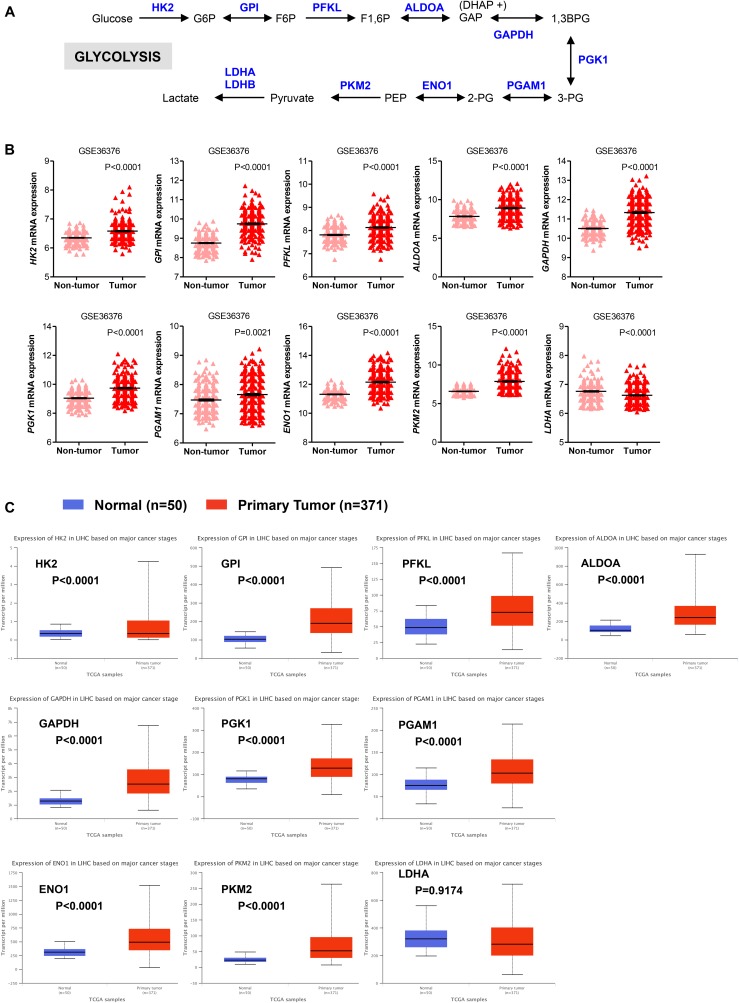
Glycolytic genes are overexpressed in HCC. **(A)** A simplified representation depicting the glycolytic pathway in liver tumors. Abbreviations of the enzymes are as follows: hexokinase 2 (HK2), glucose-6-phosphate isomerase (GPI), phosphofructokinase liver isoform (PFKL), aldolase A (ALDOA), glyceraldehyde 3 phosphate dehydrogenase (GAPDH), phosphoglycerate kinase 1 (PGK1), phosphoglycerate mutase 1 (PGAM), enolase 1 (ENO1), and pyruvate kinase M2 (PKM2), lactate dehydrogenase (LDH). Abbreviations of the metabolites are as follow: glucose-6-phosphate (G6P), fructose-6-phosphate (F6P), fructose 1,6-biphosphate (F1,6BP), glyceraldehyde 3-phosphate (GAP), and dihydroxyacetone phosphate (DHAP), 1,3-biphosphoglycerate (1,3BPG), glycerol-3-phosphate (3-PG), glycerol-2-phosphate (2-PG), phosphoenolpyruvate (PEP). **(B)** Scatterplots showing the transcript levels of different glycolytic enzymes in the clinical data set GSE36376 consisting of HCC (*n* = 240) and adjacent non-tumor (*n* = 193) liver tissue (Lim et al., 2013). The horizontal lines indicate mean ± SEM *P*-values were calculated by non-parametric Mann–Whitney tests. **(C)** Boxplots showing differential gene expression of glycolytic enzymes among normal liver tissues (*n* = 50) vs. primary tumor tissues (*n* = 371) (TGA-LIHC samples) analyzed using the UALCAN bioinformatic tool of genomic database (Ally et al., 2017; Chandrashekar et al., 2017). Values are expressed as transcript per million. For each box plot, the whiskers represent the 2.5–97.5th percentile range of values, the lower and up boundaries denote the 25th and the 75th percentile of each data set, respectively, and the horizontal line represents the median value for each group. *P*-values were calculated by *t*-test.

The glycolytic pathway consists of ten enzymatic reactions through which glucose is converted into pyruvate. Among the multiple isoforms of enzymes that catalyze each glycolytic reaction, we evaluated mRNA expression levels of those that are predominantly expressed in the liver (**Figure 1A**; Gatenby and Gillies, 2004; Lunt and Vander Heiden, 2011; Hay, 2016). As shown in **Figure 1B**, expression of the majority of glycolytic transcripts, including the rate limiting glycolytic enzyme HK2 [hexokinase 2], ALDOA [aldolase, fructose-bisphosphate A], PFKL [6-phosphofructokinase, liver type], GAPDH [glyceraldehyde 3-phosphate dehydrogenase], PKM2 [pyruvate kinase M2]) was significantly increased in HCC livers compared to their adjacent non-tumor tissues (*P* < 0.0001). An exception was the expression of PGAM1 transcripts that was still significantly higher (*P* = 0.0021) but with a less extent compared to other enzymes. LDHA [lactate dehydrogenase A] enzyme converts pyruvate into lactate in a reaction that generates NAD+, diverting glycolysis-derived pyruvate from the mitochondrial oxidative pathway (Gatenby and Gillies, 2004; Hay, 2016). As such, the decreased expression and activity of LDHA would favor the routing of pyruvate into mitochondria where it can be further metabolized through TCA and oxidative phosphorylation. Expression of LDHA was significantly reduced in HCC samples compared to their adjacent non-tumor tissues. In contrast, mRNA expression of the other lactate dehydrogenase isoform, LDHB, showed no significant changes (*P* = 0.0797; data not shown). Similar results were also obtained in The Cancer Genome Atlas (TCGA) dataset consisting of 371 primary HCC tumors and 50 normal liver samples (LIHC cohort) (Ally et al., 2017). We observed significantly higher expression of all glycolytic enzymes in primary HCC samples compared with normal livers; the only exceptions were LDHA and LDHB (**Figure 1C** and data not shown).

**FIGURE 2:**
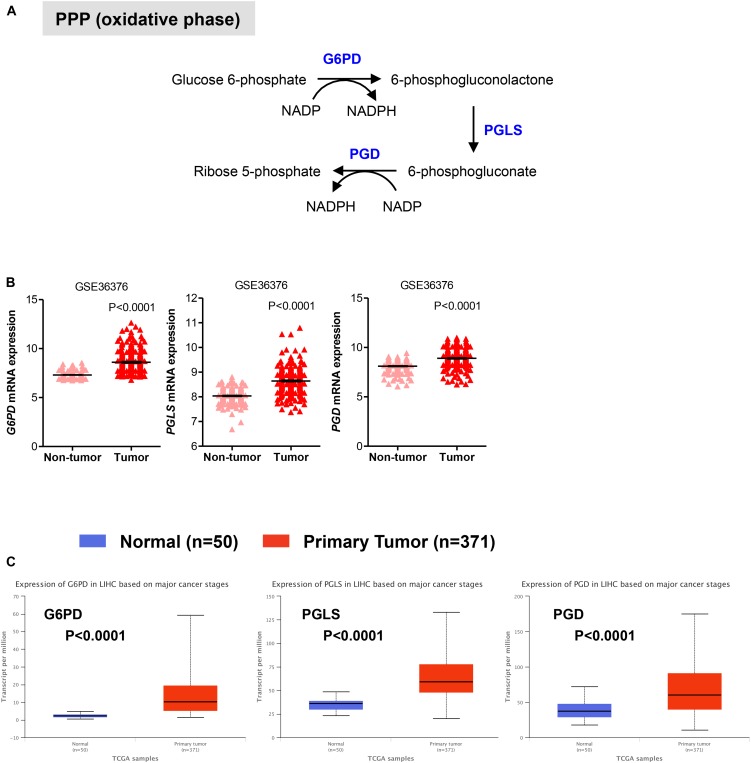
Expression of genes in pentose phosphate pathway (PPP). **(A)** Diagram of the oxidative phase of the PPP. Abbreviations of the enzymes are as follows: glucose-6-phosphate dehydrogenase (G6PD), 6-phosphogluconolactonase (PGLS), 6-phosphogluconate dehydrogenase (PGD). Activation of the two dehydrogenase enzymes, G6PD – the rate-limiting enzyme – and PGD, results in the production of NADPH, H+ ions, and ribose 5-phosphate. **(B,C)** Gene expression analyses showing enhanced expression of G6PD, PGLS, and PGD in primary HCC tumor samples compared to either adjacent non-tumor samples in GSE36376 data set **(B)** or normal liver tissues in TGA-LIHC data set **(C)**, respectively. *P*-values were calculated by nonparametric Mann–Whitney tests in **(A)** or by *t*-test **(B)**.

We also evaluated the mRNA levels of enzymes involved in the oxidative phase of PPP. In this phase, two molecules of NADP+ are reduced to NADPH, utilizing the energy from the conversion of glucose-6-phosphate into ribulose 5-phosphate, which then enter the non-oxidative phase leading to precursors of nucleotide synthesis (**Figure 2A**; Hay, 2016; Kowalik et al., 2017). We found that in HCC samples the transcript levels of the rate limiting enzyme glucose-6-phosphate dehydrogenase (G6PD) were significantly higher than those in adjacent non-tumor (*P* < 0.0001) and normal livers (*P* < 0.0001) samples, respectively (**Figures 2B,C**). Similar to G6PD, mRNA levels of the other two enzymes involved in the oxidative phase of the PPP, 6-phosphogluconolactonase (PGLS) and 6-phosphogluconate dehydrogenase (PGD), were higher in HCC samples compared to control tissue (**Figures 2B,C**). Altogether these analyses are consistent with an increase in glycolysis and PPP pathways, leading to sustained ATP and cellular building blocks production both needed for abnormal hepatocytes proliferation (Gatenby and Gillies, 2004; Kowalik et al., 2017).

### Gene Expression of Enzymes Involved in Mitochondrial Oxidative Metabolism in HCC

**FIGURE 3:**
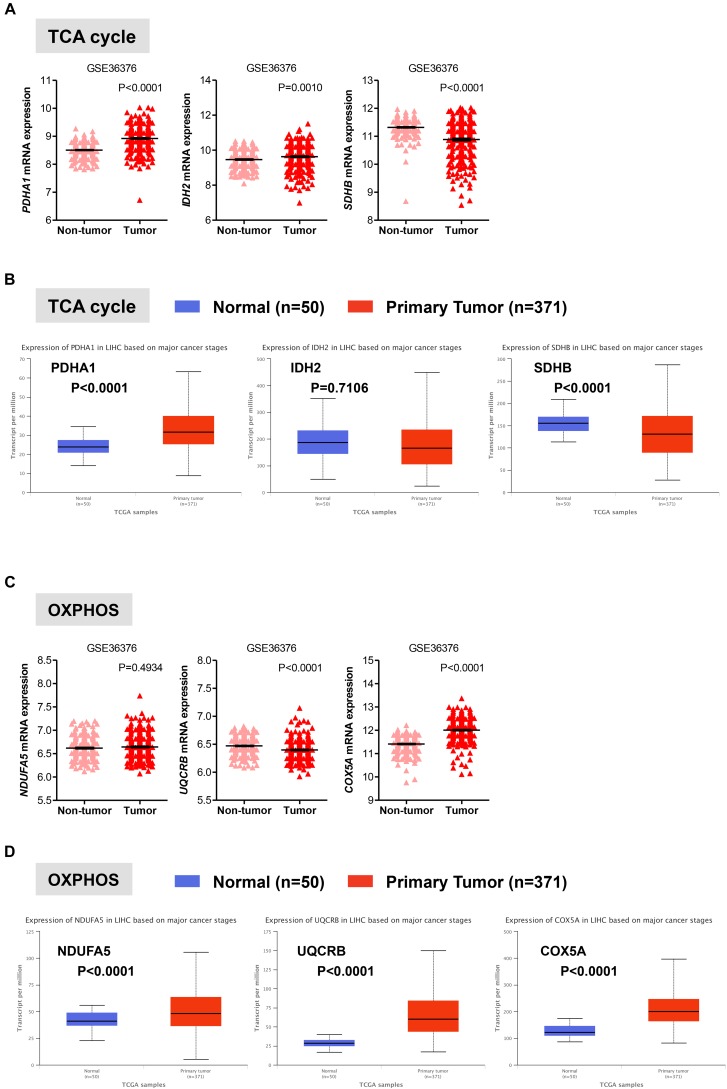
Gene expression analysis of the oxidative mitochondrial metabolism in HCC. **(A,C)** Scatterplots showing the transcript levels of representative TCA **(A)** and OXPHOS **(C)** enzymes in the clinical data set GSE36376 consisting of HCC (*n* = 240) and adjacent non-tumor (*n* = 193) liver tissue (Lim et al., 2013). The horizontal lines indicate mean ± SEM *P*-values were calculated by nonparametric Mann–Whitney tests. **(B,D)** Boxplots showing differential gene expression of TCA **(B)** and OXPHOS **(D)** among normal liver tissues (*n* = 50) vs. primary tumor tissues (*n* = 371) (TGA-LIHC samples) analyzed using the UALCAN bioinformatic tool of genomic database (Ally et al., 2017; Chandrashekar et al., 2017). Values are expressed as transcript per million. For each box plot, the whiskers represent the 2.5–97.5th percentile range of values, the lower and up boundaries denote the 25th and the 75th percentile of each data set, respectively, and the horizontal line represents the median value for each group. *P*-values were calculated by *t*-test.

Once in the mitochondria, pyruvate can be converted into acetyl-CoA by the pyruvate dehydrogenase (PDH) complex. Acetyl-CoA then enters into the TCA cycle to generate NADH and FADH2, which transfer their electrons to the electron transport chain to generate ATP through OXPHOS (Lunt and Vander Heiden, 2011; Ahn and Metallo, 2015; Liberti and Locasale, 2016). In two distinct data sets, we observed that in HCC liver samples the expression of PDHA1 gene, which encodes the E1 alpha 1 subunit of the PDH complex, was significantly higher than that in non-tumor tissue (*P* < 0.0001) or normal livers (*P* < 0.0001), while in HCC samples the expression of the succinate dehydrogenase (SDHB), which converts succinate into fumarate in the TCA, was significantly lower than that in non-tumor tissue (*P* < 0.0001) or normal livers (*P* < 0.0001) (**Figures 3A,B**). These observations are consistent with a recent study showing that decreased expression levels of SDHB in HCC promote the Warburg effect (Tseng et al., 2018). Less clear was the gene expression pattern of the isocitrate dehydrogenases 2 (IDH2), which converts isocitrate to alpha-ketoglutarate in the TCA. Expression levels of IDH2 were higher in HCC samples compared to adjacent non-tumor samples (*P* = 0.0010) from the GSE36376 data set, while IDH2 expression showed no changes in the TGA-LIHC data set (*P* = 0.7106) (**Figures 3A,B**).

Oxidative phosphorylation is coupled with the electron transport, which is organized into four large membrane-embedded proteins complexes I to IV (Alberts et al., 2002), to generate ATP. As recent studies have shown that certain types of cancer rely on both glycolytic and mitochondrial metabolism for ATP production (Lunt and Vander Heiden, 2011; Liberti and Locasale, 2016; Gentric et al., 2017), we examined whether this was the case for HCC. We analyzed the expression profile of representative genes involved in the formation of the electron transport chain complexes including NDUFA5 (complex I), UQCRB (complex III), and COXA5 (complex IV). Except for COXA5, expression of NDUFA5 and UQCRB were either constant (NDUAF5; *P* = 0.4934) or significantly reduced (UQCRB; *P* < 0.0001) in HCC when compared to adjacent non-tumor tissue in the GSE36376 cohort (**Figure 3C**). However, the expression levels of all three genes showed a significant increase in HCC samples compared to normal liver samples (**Figure 3D**).

Collectively, our results indicate that in HCC livers the expression of genes involved in the glycolytic and oxidative metabolism is higher relative to normal livers, while the expression of oxidative metabolic genes is either comparable or reduced compared to surrounding non-tumor tissue (**Figures 1–3**). Because HCC develops in the settings of cirrhosis, it is likely that surrounding non-tumor tissue is indeed a cirrhotic tissue with the presence of tumor nodules nearby (Hoshida et al., 2013). Therefore, the apparent discrepancy between the results obtained from our analysis of the two distinct data sets may reflect diverse microenvironments that differentiate a normal liver from an adjacent-to-tumor liver.

Importantly, Idle and colleagues (Beyoglu et al., 2013) have measured the expression of different tissue metabolites (including glucose, glycerol-3 and 2-phosphate, malate, alanine) in a panel of 31 HCC livers and found that in HCC there is a four-fold increase in glycolysis over mitochondrial OXPHOS compared to corresponding non-tumor liver tissue. These tissue metabolomic studies are in line with our analyses whereby changes in transcript expression of glycolysis, but not OXPHOS, in HCC are consistent with a Warburg-type metabolism.

### Expression of Glycolysis and PPP Enzymes Correlates With Poor Patient Survival in HCC

**FIGURE 4:**
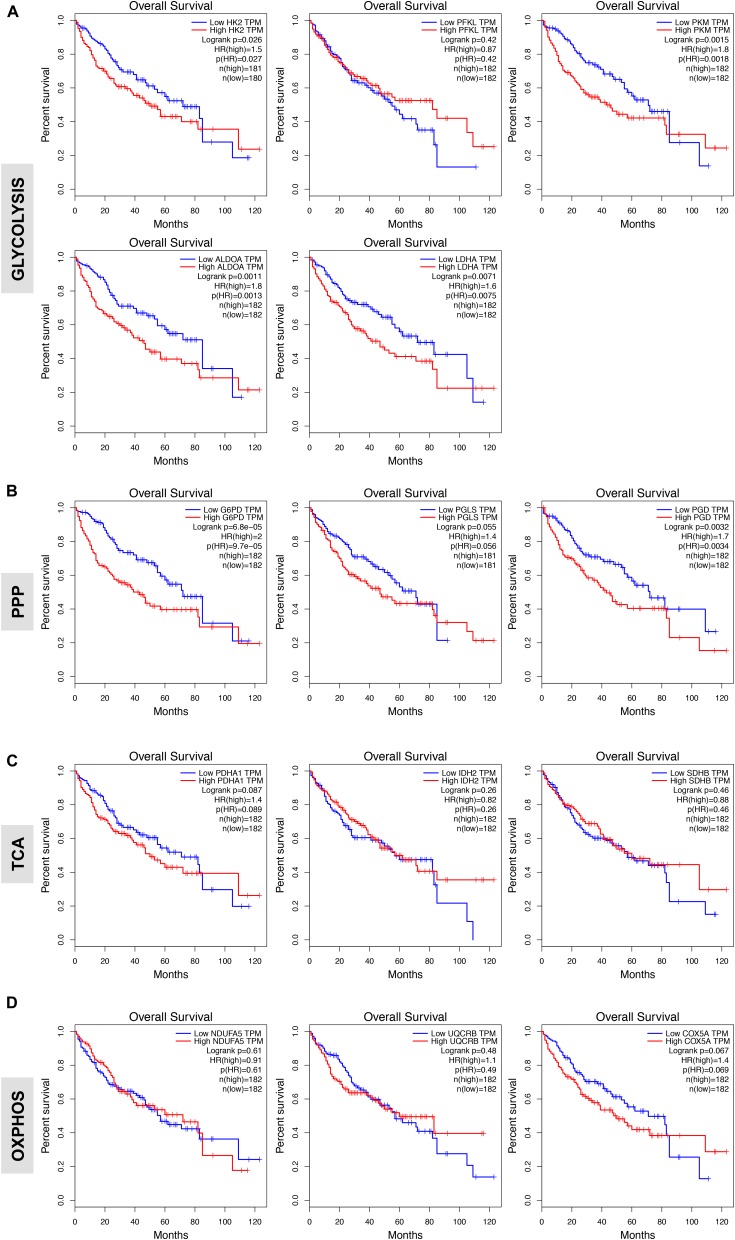
Glycolytic gene expression is associated with poor patient prognosis. **(A,B)** High expression of genes associated with glycolysis and oxidative phase of PPP significantly correlates with poor overall patients’ survival. Shown are the Kaplan-Meier overall survival curves of HCC patients according to the designated gene expression levels above or below the median value based on TGA-LIHC data set and analyzed with the GEPIA bioinformatic tool of genomic database (Ally et al., 2017; Tang et al., 2017). **(C,D)** Kaplan-Meier overall survival curves of HCC patients in TGA-LIHC data set showing homogenous prognostic significance of TCA and OXPHOS genes.

We next evaluated the prognostic value of key regulators of aerobic glycolysis and mitochondrial metabolism in HCC dataset (TCGA-LIHC) through the online tool GEPIA, a web server for cancer and normal gene expression profiling and interactive analyses (Tang et al., 2017). The cumulative patient survival curves were investigated using the Kaplan-Meier method, and differences in survival times were calculated according to the log-rank test. As shown in **Figure 4A**, when HCC patients were divided into two groups according to the median value of each gene transcript that is HK2, PKM2, ALDOA, and LDHA we found that the overall survival rate was significantly lower in the high expression groups in the TCGA-LIHC patient cohort (*P* < 0.05). Similar trends were also detected for other glycolytic genes with some reaching sufficient significance (*P* < 0.05; i.e., GAPDH, PGK1, ENO1) and very few not reaching significance (GPI, *P* = 0.100; PGAM1, *P* = 0.052; LDHB, *P* = 0.058) (data not shown). On the contrary, PFKL follows an inverse trend. High expression of PFKL was, indeed, inversely associated with poor overall survival, but this was not statistically significant (*P* = 0.420) (**Figure 4A**). Moreover, although in our differential gene expression analyses LDHA mRNA expression was not sufficiently higher in HCC livers compared to healthy and surrounding non-tumor livers (**Figures 1B,C**), it seems that LDHA expression has a prognostic value in HCC (**Figure 4A**). This is in agreement with previous studies demonstrating that high level of serum LDH is associated with poor patient overall survival in several malignancies, including HCC (Wulaningsih et al., 2015).

In examining the individual genes functioning in the oxidative phase of the PPP, we found that the overall survival rate was significantly lower in the group of patients that expressed high levels of G6PD, the rate-limiting enzyme of the PPP, and PGD mRNA (**Figure 4B**). Of note, although the expression of PGLS had no prognostic value (*P* = 0.055; **Figure 4B**), it seems that at least the rate-limiting enzyme G6PD of the oxidative phase of PPP has important clinical implications for patients with HCC. Surprisingly, metabolic genes involved in mitochondrial metabolism have no prognostic value in HCC as neither the expression of genes related to TCA cycle nor OXPHOS was associated with poor patient overall survival (**Figures 4C,D**) despite their significant increases in HCC samples compared to normal livers (**Figure 3**). This could indicate that TCA and OXPHOS enzymes may be subject to a strict post-translational regulation that uncouple the mRNA levels from the actual enzyme function in HCC livers.

Interestingly, similar trend was obtained by analyzing survival of HCC patients by using a distinct data set via the KM plotter web server tool (Szász et al., 2016) (**Supplementary Figure S1**). Overall these data indicate that glycolytic genes have prognostic role in HCC.

### Gene Expression of Glycolysis and PPP Enzymes in Cirrhotic Livers

**FIGURE 5:**
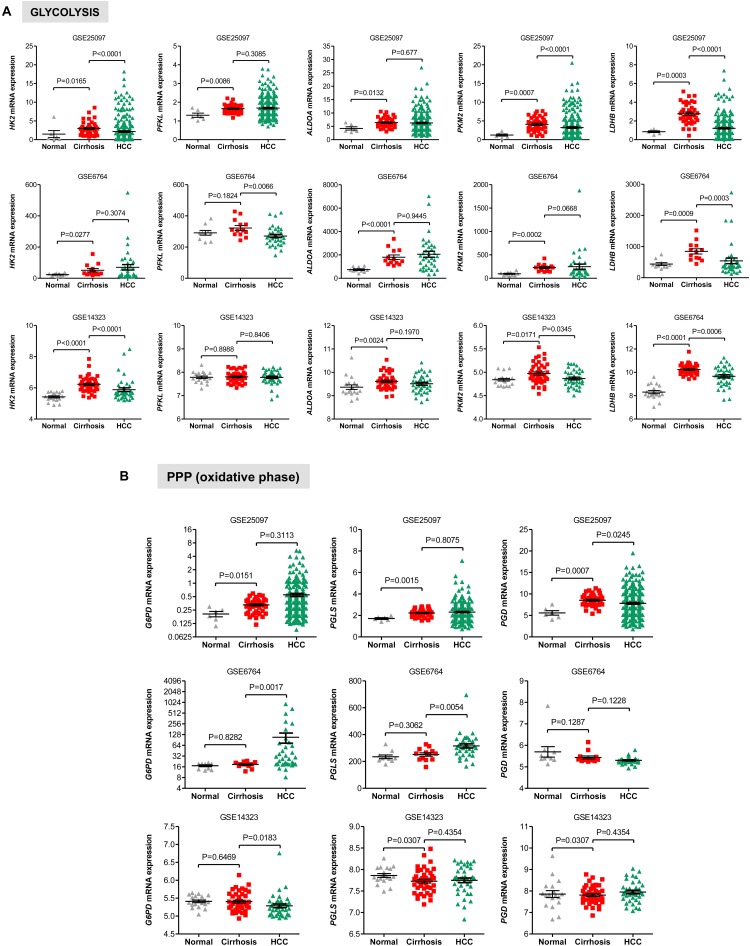
Gene expression of glycolysis and PPP enzymes in cirrhotic and HCC livers. **(A)** Scatterplots showing the increased expression of transcript levels of representative glycolytic enzymes in cirrhotic livers compared to HCC livers and normal healthy liver tissues in three independent clinical data sets GSE25097, GSE6764, and GSE14323 (Wurmbach et al., 2007; Sung et al., 2012; Levy et al., 2016). **(B)** Scatterplots showing differential gene expression of genes related to oxidative PPP among cirrhotic livers compared to HCC livers and normal healthy liver tissues in three independent clinical data sets GSE25097, GSE6764, and GSE14323 (Wurmbach et al., 2007; Sung et al., 2012; Levy et al., 2016). The horizontal lines indicate mean ± SEM *P*-values were calculated by non-parametric Mann–Whitney tests.

Liver cirrhosis is a precancerous state of HCC (Wright, 1991; Borzio et al., 1995; Donato et al., 2001; El-Serag, 2011; Jemal et al., 2011; Forner et al., 2018). Therefore, cirrhosis is a unique model for investigating markers for early detection of HCC *in vivo* (El-Serag, 2011; Forner et al., 2018; Fujiwara et al., 2018). To better understand the metabolic alterations in early liver carcinogenesis, we examined the expression of glycolytic transcripts as well as transcripts of mitochondrial metabolism in three independent clinical data sets totalising 94 cirrhotic livers and 34 healthy livers. We surveyed the transcripts levels of representative glycolysis enzymes. As shown in **Figure 5A**, statistically significant differences in mRNA levels were detected between cirrhotic and normal livers for HK2, ALDOA, and PKM2 in the three data sets analyzed. As LDHA probe was not found in the GSE25097 cohort, we analyzed the expression of LDHB and found that (like the other glycolytic enzymes) expression of LDHB was also increased in cirrhotic liver samples compared to normal livers (**Figure 5A**). On the contrary, expression levels of PFKL were significantly higher in cirrhotic livers compared to healthy specimens in the GSE25097 cohort (*P* = 0.0086) but found constant in the two other data sets (*P* = 0.1824 and *P* = 0.9888). Collectively, these results are consistent with those observed in HCC. Indeed, while the expression of HK2, ALDOA, and PKM2 transcripts is increased in HCC compared to normal livers and associated with poor overall patient survival, elevated levels of PFKL scored in HCC had no prognostic value (see **Figure 4**). This suggests that the expression of these genes in cirrhotic livers resemble the gene expression signature of HCC (**Figure 5A**).

**FIGURE 6:**
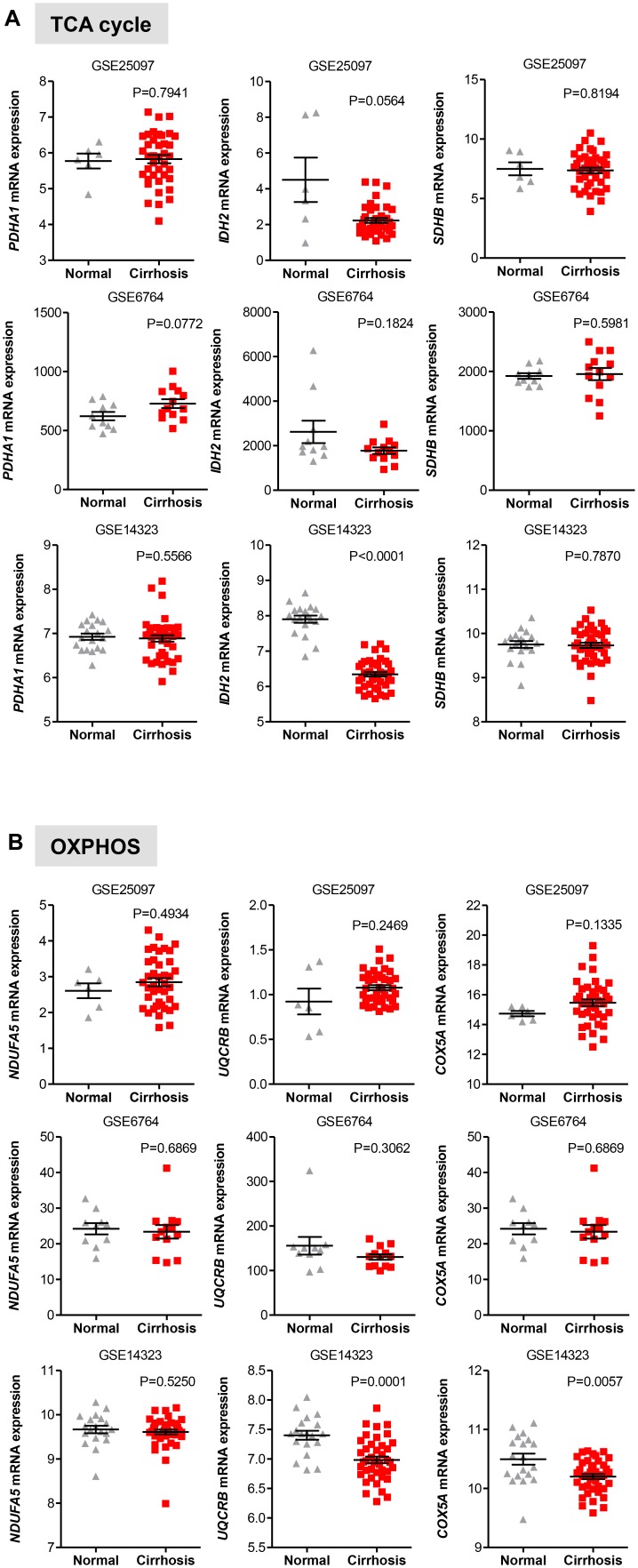
Gene expression of TCA and OXPHOS enzymes in cirrhotic livers. **(A,B)** Scatterplots showing homogenous expression of representative TCA and OXPHOS biomarkers in cirrhotic livers compared to normal healthy liver tissues in three independent clinical data sets GSE25097, GSE6764, and GSE14323 (Wurmbach et al., 2007; Sung et al., 2012; Levy et al., 2016). The horizontal lines indicate mean ± SEM *P*-values were calculated by non-parametric Mann–Whitney tests.

We also evaluated the levels of transcripts encoding genes in the oxidative phase of PPP and found that while in the GSE25097 dataset there was a significant increase of the three PPP genes analyzed (including the rate limiting enzymes G6PD), no changes were observed in the other two data sets, except for PGLS, whose expression was significantly lower in cirrhotic livers (**Figure 5B**). Notably, no significant increase of glycolytic and PPP gene expression has been observed in HCC compared to cirrhotic livers (**Figures 5A,B**), suggesting that reprogramming of glucose metabolism may occur at pre-cancerous stages. For the TCA enzymes, the mRNA levels of PDHA1, IDH2, and SHDB were constant in both data sets of cirrhotic livers compared to normal livers (**Figure 6A**). Similarly, transcripts levels of representative OXPHOS genes that were highly expressed in HCC were not found differentially expressed in cirrhotic livers compared to healthy livers (**Figure 6B**). Collectively, these results suggest that, in contrast to a general increase in glycolysis genes, TCA and OXPHOS genes remained at the same level in cirrhosis. These observations suggest a shift toward aerobic glycolysis and PPP relative to oxidative phosphorylation in cirrhotic livers. This is also supported by the fact that, in contrast to HCC (**Figure 1**), cirrhotic livers exhibit elevated expression of LDHB compared to healthy livers (**Figure 5A**), suggesting that pyruvate may be diverted away from the mitochondria for ATP production.

Furthermore, as documented for HCC, Nishikawa et al. (2014) demonstrated that hepatocytes isolated from liver rats with early signs of cirrhosis shows a metabolic shift from OXPHOS to glycolysis for the production of ATP, while normal rat hepatocytes continue to use OXPHOS for ATP generation. It was also shown that expression of glycolytic genes is severely decreased in cirrhotic hepatocytes with decompensated liver function, suggesting that failing livers do not require glycolysis to overcome early sign of injury. Altogether these studies (Beyoglu et al., 2013; Nishikawa et al., 2014) are in line with our analyses whereby an increased expression of glycolysis transcripts, but not OXPHOS transcripts, is detected in both HCC and cirrhotic livers compared to normal livers.

### Glycolytic Gene Expression in Cirrhotic Livers Is Associated With the Risk of Developing HCC

**FIGURE 7:**
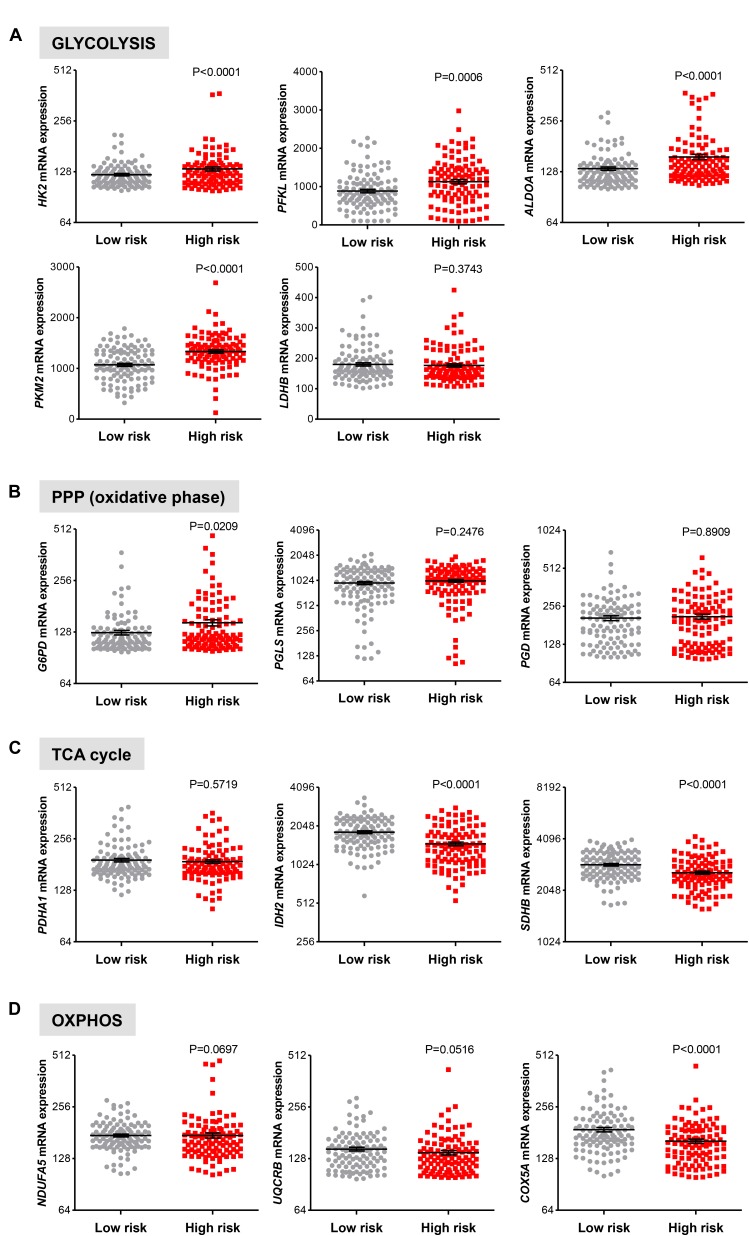
Increased risk of developing HCC in cirrhotic patients significantly correlates with high expression of glycolytic genes. **(A–D)** Levels of transcripts in the clinical data set GSE15654 consisting of 115 patients with newly diagnosed cirrhosis who were prospectively followed up in an HCC surveillance program and classified as having low (*n* = 55) and high (*n* = 60) HCC risk based on the rates of patient survival and risk of developing HCC (Hoshida et al., 2013). The horizontal lines indicate mean ± SEM *P*-values were calculated by non-parametric Mann–Whitney tests.

Next, we examined if genes involved in glycolysis, PPP, TCA cycle and OXPHOS are associated with a progression of cirrhosis to HCC and patient survival. The clinical data set GSE15654 consists of biopsies obtained from patients with hepatitis C-related Child-Pugh A cirrhosis who were prospectively followed in an HCC surveillance program for a median of 10 years and classified as low (*n* = 55) and high (*n* = 60) HCC risk based on the rates of patient survival and incidence of developing HCC (**Table 1**; Hoshida et al., 2013). We examined the expression of HK2, PFKL, ALDOA, PKM2, and LDHB transcripts by interrogating this public gene expression database. As shown in **Figure 7A** and **Table 1**, high mRNA expression of HK2, PFKL, ALDOA, and PKM2 positively correlated with a progression of cirrhosis to HCC and a worse survival rate. An exception was LDHB, which is not differentially expressed in the two prognostic groups.

**Table 1:** Summary of the clinical parameters associated with each group of cirrhotic livers.

Prognosis	Low HCC risk	High HCC risk
HCC development rate	18%	42%
Patients’ survival rate	85%	63%


For the oxidative phase of PPP enzymes, high G6PD mRNA expression levels were found to be associated with a progression of cirrhosis to HCC and reduced survival rate (*P* = 0.0209), while PGLS and PGD show no differential expression (*P* > 0.050; **Figure 7B**). Finally, in line with results showed in **Figure 6**, no increase in expression of the metabolic genes in TCA cycle and OXPHOS was observed between the low and high HCC risk groups (**Figures 7C,D**). On the contrary, the expression of IDH2, SDHB, and COXA5 were inversely correlated with poor prognosis (**Figures 7C,D**). Thus, in cirrhotic livers high expression of glycolysis genes is associated with an elevated risk of developing HCC, suggesting that the glycolytic phenotype has a possible prognostic role in cirrhotic patients. Moreover, HK2, PFKL, ALDOA, PKM2, and LDHB levels show a weak differential expression in cirrhotic and HCC livers (**Figure 5A**), indicating that the acquisition of a glycolytic phenotype occurs during both initiation and maintenance of HCC. Collectively, these results suggest that glycolytic enzymes may represent potential targets for HCC chemoprevention.

## Discussion

In more than 80% of cases HCC arise in the settings of cirrhosis (El-Serag, 2011; Jemal et al., 2011; Forner et al., 2018; Fujiwara et al., 2018). Therefore, understanding how cirrhosis develops from normal liver and progresses to HCC is particularly relevant for identifying chemoprevention therapies. Hepatitis B (HBV) or C (HCV) viral infection, chronic alcohol consumption and nonalcoholic fatty liver disease have been reported to be the prominent causes of the development of cirrhosis (El-Serag, 2011). Nevertheless, the early biochemical and molecular events that underlie the progression of cirrhosis to HCC remain largely unclear.

Notable features of malignant hepatocytes – the predominant liver parenchymal cells – include a high proliferative potential. In order to proliferate HCC hepatocytes readjust their metabolic activities to fulfill the bioenergetic and anabolic needs for doubling mass (Gatenby and Gillies, 2004; Lunt and Vander Heiden, 2011; Hay, 2016). It is, however, unclear how and when HCC cells acquire these metabolic changes during the hepatocarcinogenetic process.

Here we report changes in transcript expression of glycolysis and PPP genes in cirrhosis consistent with a Warburg-type metabolism and show a positive correlation between glycolytic gene expression and the risk of developing HCC and poor patient survival.

**FIGURE 8:**
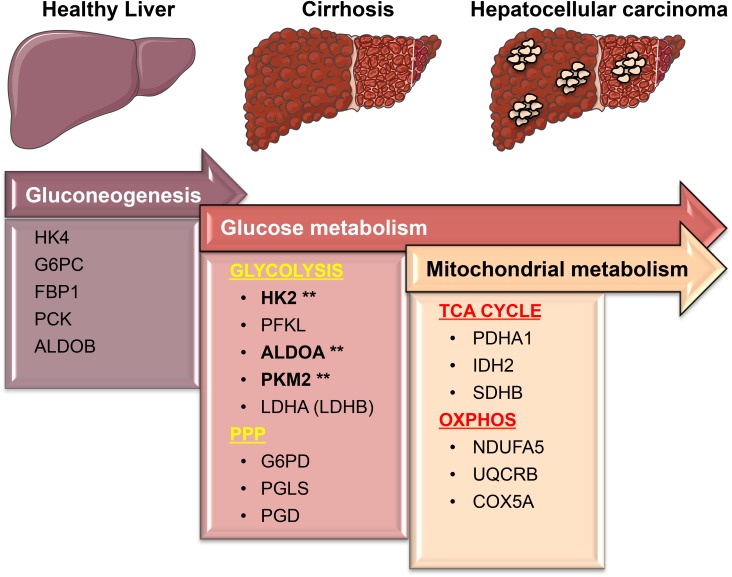
A simplified model showing transcriptional changes in gene expression of metabolic enzymes that characterize the progression from a normal liver to cirrhosis, which culminates in HCC. One of the most pronounced transcriptional changes during the transition from normal to HCC is the suppression of HK4 and ALDOB followed by the induction of the high affinity HK2 and ALDOA. This allows glucose to enter in the cells and be converted into pyruvate. PKM2 and lactate dehydrogenase (LDHA/LDHB), the last two enzymes in the glycolytic pathway, are essentially needed to maintain the high glycolytic rate of liver carcinomas by fast removal of accumulating upstream intermediates to favor the entrance into other metabolic pathways such as the oxidative phase of PPP. Surprisingly, those metabolic changes mostly occur early on the premalignant stage (cirrhosis) during hepatocarcinogenesis. While in cirrhotic livers glycolysis provides both biosynthetic building blocks and anti-oxidant defenses needed for the survival (as opposed to apoptosis) of damaged cells, in livers with HCC glycolysis cooperates with OXPHOS and TCA cycle to sustain energy needed for fast proliferation and metastasis. Our analyses suggest that the expression of glycolytic enzymes such as HK2, ALDOA, and PKM2 (marked by asterisks) in precancerous, chronically inflamed livers may be considered as new predictive biomarkers to prevent the development of liver cancer. Figure was created modifying illustrations provided by Servier (http://smart.servier.com) under Creative Commons Attribution 3.0 Unported License.

Compared to normal livers, elevated mRNA expression of key metabolic genes in glycolysis, PPP, TCA, and OXPHOS were found in livers samples from patients with HCC, implying a pathogenic role of glucose metabolism for this disease. A distinctive function of normal hepatocytes is the conversion of non-carbohydrate carbon substrates, like lactate and pyruvate, into glucose through the gluconeogenesis pathway. Several gluconeogenic enzymes such as glucose-6-phosphatase (G6Pase), fructose-1,6-bisphosphatase (FBP1), and PEP carboxykinase (PEPCK) are known to bypass the first, second and third irreversible reactions of the glycolytic pathway, respectively, resulting in slowing down the rate of glycolysis in normal hepatocytes (**Figure 8**; Hay, 2016). In HCC, G6Pase, FBP1, and PEPCK, together with the low-affinity hexokinase 4 (HK4) are suppressed, while the high-affinity hexokinase HK2 is overexpressed (Wang et al., 2012; Hay, 2016). Consistent with this, we found that HK2 is not only highly expressed in HCC but also in cirrhosis as compared to normal liver samples, suggesting the occurrence of the glycolytic phenotype early in the hepatocarcinogenetic process. This hypothesis is supported by the observation that the expression levels of other key glycolytic enzymes such as ALDOA and PKM2 are also overexpressed in cirrhotic livers. Among the three known isoforms of the glycolytic enzyme aldolase (ALDOA, ALDOB, and ALDOC), which catalyzes the reversible conversion of Fructose 1,6-bisphosphate (F1,6BP) to Dihydroxyacetone phosphate (DHAP) and Glyceraldehyde 3-phosphate (G3P), ALDOA catalyzes this reaction more efficiently thus accelerating the flux through glycolysis (Castaldo et al., 2000; Hay, 2016). Indeed, ALDOA was reported to be the main isoform expressed in most cancer types (Asaka et al., 1994). Our finding that the levels of ALDOA are up regulated in cirrhotic and HCC samples suggests that a high glycolytic phenotype is a remarkable feature of both precancerous and cancerous conditions.

Recent works have established that the pyruvate kinase M2 isoform (PKM2), which catalyzes the synthesis of pyruvate and ATP, using phosphoenolpyruvate (PEP) and ADP as substrates, is a critical driver of the Warburg effect (Christofk et al., 2008; Cortés-Cros et al., 2013; Israelsen et al., 2013; reviewed in Wiese and Hitosugi, 2018). Indeed, similar to other types of cancer cells, HCC cells express PKM2, the pyruvate activity (PK) of which is maintained at low levels by pro-survival signaling pathways (reviewed in Papa and Bubici, 2016). The low activity of PKM2 allows the accumulation of glycolytic intermediates that can be diverted into the biosynthetic pathways to form amino acids, nucleic acids, and lipids (Christofk et al., 2008; Cortés-Cros et al., 2013; Israelsen et al., 2013). While promoting these biosynthetic pathways, low PKM2 activity also contributes to boost the levels of reduced nicotinamide adenine dinucleotide phosphate (NADPH) and antioxidant reduced glutathione (GSH), which serves to detoxify reactive oxygen species (ROS) whose accumulation would result in apoptosis in chronically injured and tumor tissues (Papa and Bubici, 2016).

We indeed observed remarkable higher expression levels of PKM2 in cirrhotic and HCC livers compared to normal livers. Thus, although further studies are required, it is reasonable to hypothesize that the enhanced expression of PKM2 in cirrhotic livers may contribute to the apoptosis resistance of damaged (injured) hepatocytes that continue to survive, proliferate and accumulate until becoming malignant HCCs. Of note is also the observation that the expression of LDHA (or LDHB) (the glycolytic enzymes converting pyruvate in lactate) have no HCC risk associated in cirrhotic patients (**Figure 7**), suggesting that glycolytic-derived pyruvate may not be all converted in lactate and is therefore channeled to other metabolic pathways (i.e., mevalonate pathway; discussed below).

While it was long believed that the aerobic glycolytic phenotype is associated with an impaired mitochondrial oxidative metabolism (Lunt and Vander Heiden, 2011), recent research in the field have demonstrated that glycolytic and mitochondrial metabolism are both used by cancer cells for ATP production and macromolecule synthesis (Weinberg et al., 2010; Ahn and Metallo, 2015; Gentric et al., 2017; Takahashi et al., 2018). In agreement with these studies, our analysis in HCC samples show high expression levels of genes related to mitochondrial metabolism compared to normal livers. The fact that in cirrhosis the expression levels of TCA and OXPHOS genes are lower than those in normal livers or remain unchanged suggests the occurrence of both glycolytic and mitochondrial metabolism in HCC only. By contrast, cirrhotic livers have high expression of HK2, ALDOA, PKM2, and LDHB as well as display a significant increase in G6PD expression. It appears that metabolic readjustments (that is, glycolysis shift) occur in cirrhosis.

This may actually be explained by the fact that chronically injured tissues such as cirrhotic livers have a more natural tendency to trigger protective programs against ROS-inflicted cell death (via necrosis or apoptosis), rather than mitochondrial metabolism that contributes to the generation of ROS (Papa et al., 2009; Luedde et al., 2014; DeBerardinis and Chandel, 2016).

Indeed, it has been suggested that in cancer cells the production of ROS by complex I, II, and III of the electron transport chain contributes to DNA damage and is required for cancer cell survival, Kras-induced cellular transformation and cancer metastasis (Weinberg et al., 2010; Takahashi et al., 2018; reviewed in Lunt and Vander Heiden, 2011; Reczek and Chandel, 2015; DeBerardinis and Chandel, 2016). Thus, it appears that in inflamed livers the transcriptional regulation of genes involved in glycolysis operates to trigger cellular protective programs that serve to counterbalance the chronic injury. Besides transcriptional regulation, metabolic enzymes are known to be tightly regulated by post-transcriptional modifications including phosphorylation, acetylation, glycosylation which affect the metabolic cellular activities and have not been investigated in this study. Of note, hypoxia (via activation of HIF-1α transcription factor) and other intracellular signaling pathways (i.e., PI3K/AKT, JNK, ERK, PTEN, p53) have also been shown to regulate and drive the metabolic shift toward aerobic glycolysis of HCC cells (Iansante et al., 2015; Cassim et al., 2018). It should be instructive to evaluate the differential signature of these metabolic regulators in normal and cirrhotic livers and test whether these pathways are activated in cirrhotic livers. Further studies such as protein expression analysis are therefore required to understand the specific mechanisms underlying the metabolic changes observed in HCC and cirrhosis, although it might be difficult due to shortage of human cirrhotic samples available.

Prevention of HCC is an unmet medical need (El-Serag, 2011; Jemal et al., 2011; Forner et al., 2018). Interestingly, HK2, ALDOA and PKM2 expression levels appear to have important clinical implication for patients with cirrhosis, as analysis of cirrhotic livers from patients followed up during a span of 10 years showed a positive correlation between high expression of glycolysis genes and progression of cirrhosis to HCC. This suggests that expression of these glycolytic enzymes could be used as a new biomarker for the risk of developing HCC. By contrast, neither TCA nor OXPHOS gene expression analyzed in this study was associated with an increased risk of HCC.

Although there are potential limitations associated with the nature of the analyses carried out in this study, we sought to detect an early sign of metabolic changes in the development of HCC and show that HK2, ALDOA and PKM2 expression levels in cirrhotic livers could be used as new predictive biomarkers for HCC development. Early studies demonstrated that rat cirrhotic livers show a metabolic shift from OXPHOS to glycolysis, while normal rat hepatocytes continue to use OXPHOS (Chen et al., 2012; Beyoglu et al., 2013; Nishikawa et al., 2014; Gao et al., 2015). Further support to our observations comes from ongoing clinical trials for prevention of HCC development and recurrence. Randomized clinical trials are currently ongoing to explore the use of statins (i.e., Simvastatin, Atorvastatin) to prevent either HCC development in cirrhotic (precancerous) patients or HCC recurrence in HCC-free patients surgically treated with curative ablation or hepatectomy (Goodman, 2016; Chen, 2017; Fujiwara et al., 2018). Statins are a class of lipid-lowering medications that target the rate-controlling enzyme (NADH/NADPH-dependent) 3-hydroxy-3-methyl-glutaryl-coenzyme A (HMG-CoA) reductase of the mevalonate pathway in which cholesterol and other isoprenoids are produced (Clendening and Penn, 2012; Fujiwara et al., 2018). The mevalonate pathway begins with acetyl-CoA and ends with the production of isopentenyl pyrophosphate (IPP) and dimethylallyl pyrophosphate (DMAPP), precursors of isoprenoids. Importantly, it has been shown that aerobic glycolysis positively regulates the mevalonate pathway by producing pyruvate, which is needed for the initial formation of acetyl-CoA (Clendening and Penn, 2012). Thus, it is very likely that by blocking the mevalonate pathway via inhibition of HMG-CoA reductase, statins may contribute to the suppression of aerobic glycolysis through a negative feedback (Clendening and Penn, 2012). If these clinical trials would be beneficial to reduce HCC risk, further studies would be necessary to directly link the mevalonate pathways to aerobic glycolysis in liver cirrhosis.

## Ethics Statement

The study involves the analyses of public available data sets using human subjects. Therefore, approval for the use of human subject has been granted to third parties that have deposited the public data sets, which have been properly cited in the text.

## Author Contributions

NL, MC, CB, and SP performed the experiments. CB and SP are both senior authors, conceived the idea, chiefly carried out data analysis and interpretation, coordinate the study, and wrote the manuscript, which was commented by all the authors.

## Conflict of Interest Statement

The authors declare that the research was conducted in the absence of any commercial or financial relationships that could be construed as a potential conflict of interest.
