# Supplementary material for: High Expression of Glycolytic Genes in Cirrhosis Correlates With the Risk of Developing Liver Cancer
Source: Front Cell Dev Biol. 2018 Oct 31;6:138. doi: 10.3389/fcell.2018.00138 (PMC6220322; doi:10.3389/fcell.2018.00138)
Supplement: Supplementary file 1 [file Image_1.pdf]

Supplemental Figure 1

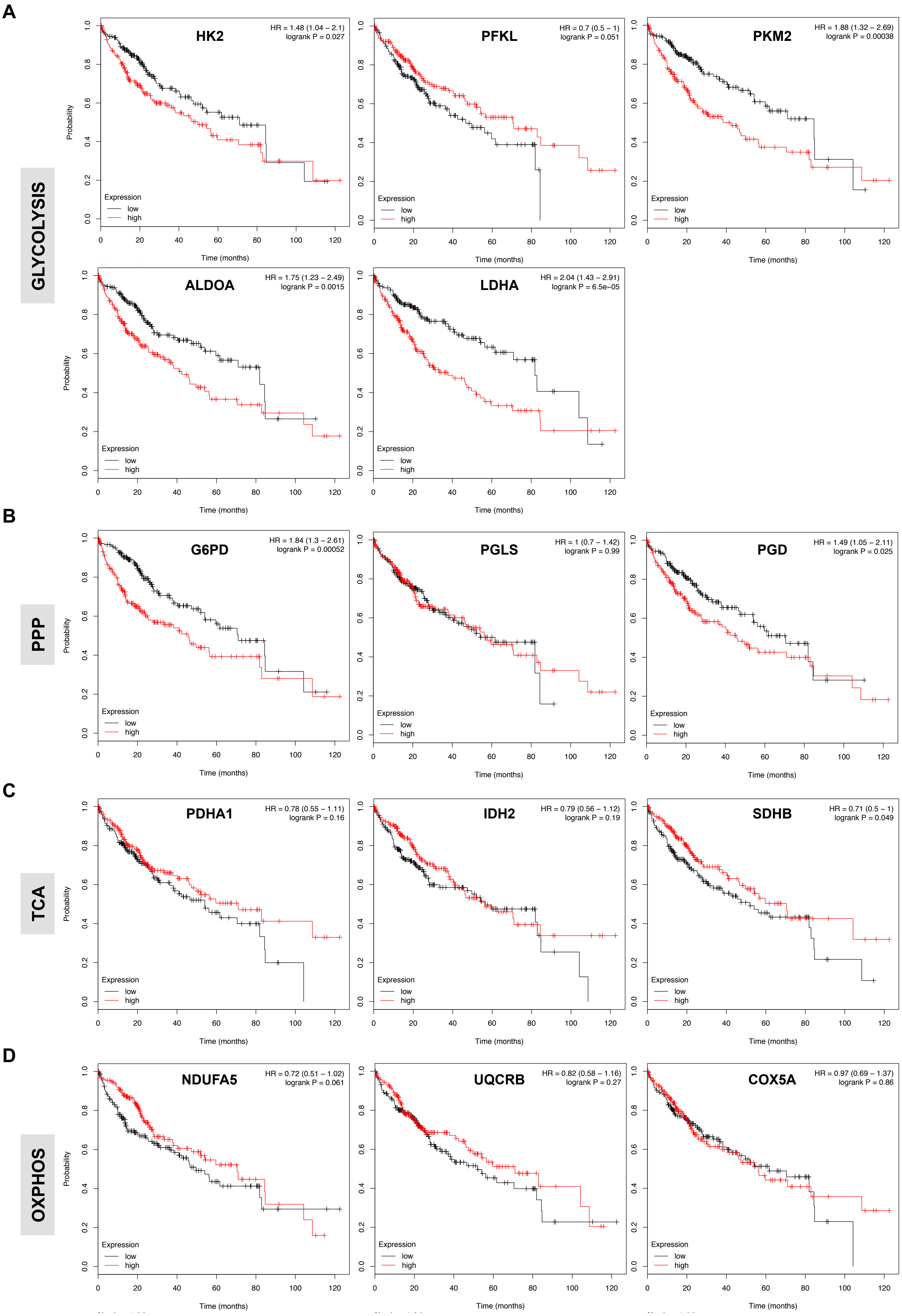

Supplemental Figure 1. High expression of genes associated with glycolysis correlates with poor prognosis. **A-D)** Shown are the Kaplan-Meier overall survival curves of HCC patients according to the designated gene expression levels above or below the median value based on liver hepatocellular carcinoma data set analysed with the KMplotter bioinformatic tool of genomic database (*Szász AM, et al. 2016*).
